# Neutralizing a Springboard for Inflammation: Physical Activity to Control the Immune Network

**DOI:** 10.3390/healthcare9091196

**Published:** 2021-09-10

**Authors:** Victor Kallen, Rogier Scherder, Maarten J. Cramer, Jacqueline Stam, Bruce Johnson, Erik Scherder

**Affiliations:** 1 The Netherlands Organization for Applied Sciences (TNO), P.O. Box 23, 3769 ZG Soesterberg, The Netherlands; Jacqueline.stam@tno.nl; 2 Department of Sports Medicine, University Medical Center Utrecht, Heidelberglaan 8, 3584 CS Utrecht, The Netherlands; R.J.Scherder-3@umcutrecht.nl; 3 Department of Cardiology, University Medical Center Utrecht, Heidelberglaan 8, 3584 CS Utrecht, The Netherlands; M.J.M.Cramer@umcutrecht.nl; 4 Department of Cardiovascular Medicine, Mayo Clinic, 200 First St. SW, Rochester, MN 55905, USA; johnson.bruce@mayo.edu; 5 Department of Clinical Neuropsychology, Vrije Universiteit Amsterdam, De Boelelaan 1105, 1081 HV Amsterdam, The Netherlands; eja.scherder@vu.nl

**Keywords:** COVID-19, exercise, stress, immune functioning, inflammation, sedentary, lifestyle

## Abstract

The severe consequences of the present Corona Virus Disease 2019 COVID-19 pandemic seem to be closely related to an already ongoing (‘first’) pandemic, directly associated with a sedentary lifestyle. It seems evident that the prognosis after infection is substantially worse for individuals suffering from, for example, (visceral) obesity, cardiovascular disease, and diabetes type 2. Consequently, it may be considered highly relevant to gain insight into the potential beneficial effects of exercise interventions to strengthen the immune system, particularly in high-risk populations. For this reason, the exercise protocols that are suggested to strengthen the immune system, which can be executed by all ages and almost all physical conditions, were reviewed and evaluated. Previously published protocols range from interrupting prolonged sitting, to regular low-to-moderate exercise activities, to high intensity, typically interval, sports formats. Reported positive effects on immune functioning appear to be induced either directly or via beneficial metabolic and/or psychological effects and become measurable after 3 weeks to 3 months. Based in these findings, it appeared possible to design an optimal exercise protocol to maximize effects on immune functioning that should be executable for all, even under restricted (‘lockdown’) circumstances.

## 1. Introduction

Directly related to the ongoing COVID-19 pandemic, an intriguing series of papers has been published highlighting the parallel existence of actually two pandemics that in a poisonous interaction appear to extend each other’s negative consequences [1,2,3]. Next to the relatively new inflammatory, COVID-19, pandemic, the other ongoing pandemic is identified as ‘physical inactivity’, or sedentary lifestyle related disease. The latter has been addressed continuously since it was more or less formally introduced as ‘pandemic’ in 2012 [4]. Data convincingly show that each year, approximately 5.3 million people die of non-communicable diseases associated with a sedentary life style, such as diabetes type 2 (T2D), obesity, and cardiovascular disease [5]. One could imagine that such a high number of deceased people would have been a wake-up call. It is alarming, however, that a 2016 update highlighted that the ‘physical inactivity’ pandemic was still overtly present, and worldwide no noticeable progress had been made in reducing the number of people with an inactive lifestyle or the prevalence of the associated morbidities. In the same year (2016), 41 million children between 0 and 5 years old were reported to suffer from overweight/obesity [6], a number that is estimated to rise to 50 million in 2030. Unfortunately, in their 2019 update Pratt and coworkers had to conclude that we failed to prioritize physical activity in a global public health plan, and that the prevalence of associated health outcomes and related mortality remain steadily increasing [7]. And now, with the emergence of the highly communicable SARS-CoV-2 virus, specifically the individuals with the same non-communicable diseases appear to be among the most vulnerable for the development of severe complications and eventually mortality after infection [8]. Consequently, it may be alarming that the worldwide policies that were put in place to counter the infection rates (‘lockdown’) typically stimulated the solitary, sedentary and inactive lifestyle that made many so vulnerable for the impact of SARS-CoV(-2) infections [1,9]. And since recent findings, e.g., in the elderly, directly linked social isolation (‘loneliness’), negative life events and depression to established cardiovascular and respiratory risk factors [10], there may even be more reason for concern. Social isolation, combined with fear of infection, might provoke stress, anxiety, frustration, and depression [11], all significantly inhibiting physical activities. Consequently, the risk for the development of pre-clinical symptoms and over time non-communicable diseases such as T2D, obesity, and cardiovascular syndromes is likely to be increased by the circumstantial incentives provided by the presently (secondary) COVID-19 pandemic and associated countermeasures (typically rather strict lockdown policies)—a toxic combination of interacting factors that will inevitably make individuals even more vulnerable for the complications associated with communicable diseases, such as COVID-19.

Although the severity of the COVID-19 pandemic might be slowly decreasing by now, this is a worrisome conclusion, as it is now well established that something relatively simple as frequently interrupting prolonged sitting and/or increasing physical activity may not only reduce the risk factors for the development of cardiovascular disease [12], but might consequently, over time, mitigate at least some of the complications following, for example, a SARS-CoV-2 infection as well [13]. That conclusion is strongly supported by a recent study including 48,440 adult patients diagnosed with COVID-19 indicating that meeting the general physical activity guidelines prior to infection is associated with a significantly reduced risk for severe COVID-19 outcomes after infection [14].

As addressed elegantly by Meyer et al. (2020), these two interventions (regularly interrupting prolonged sitting and moderate-to-vigorous ‘sports’ activities) should likely be considered as distinct behavioral strategies: associated with lockdown-related working-from-home, a considerable proportion of U.S. inhabitants reported an increase in both net sitting time and moderate-to-vigorous physical activities [15]. Importantly, however, although increasing moderate-to-vigorous exercise activities might to some extent attenuate the consequences, it does not annul all the (negative) health effects associated with extended sedentary behaviors [16,17]. Nor does it necessarily alleviate specific mental aspects associated with a sedentary lifestyle, as for example expressed in reported symptoms of fatigue [18]. Consequently, due to different effects on immune functioning, well-being and health, interrupting prolonged sitting and regularly engaging in moderate-to-vigorous physical activity seem distinct but complementary interventions.

**Figure 1:**
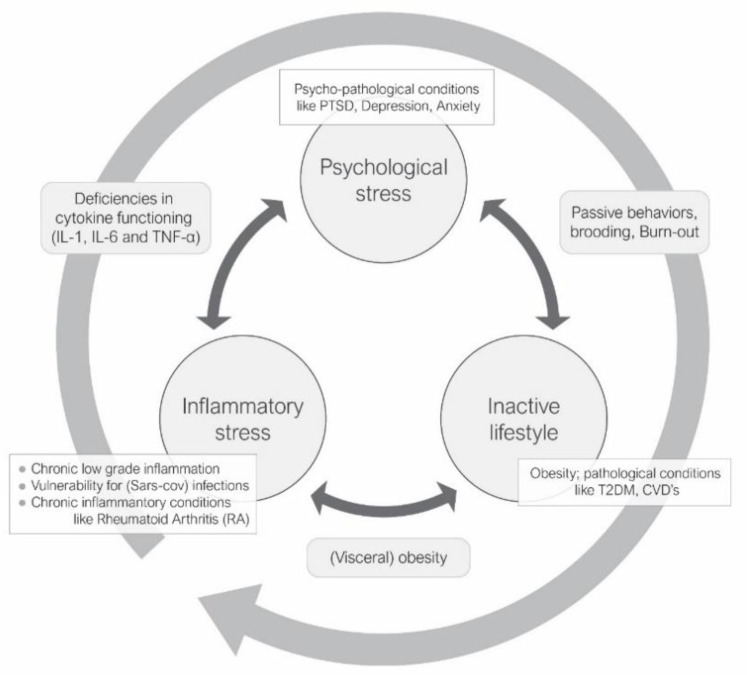
The vicious circle of an inactive/sedentary lifestyle (with associated health outcomes), psychological stress, and immune functioning. When this balance is already ‘under pressure’ (e.g., by lifestyle-related disease, or psychopathological conditions), the probability to develop complications following (Sars-Cov2) infection is likely to increase rapidly. The reduction of psychological stress and (moderate) exercise seems to be the potentially most convenient, non-invasive and effective intervention to influence this circle. Contrary to the psychological stress that lockdown policies generally induce, the latter should nevertheless be executable under such circumstances. Note: PTSD = Post Traumatic Stress Disorder, IL = Interleukin, TNF = Tumor Necrosis Factor, T2DM = Type 2 Diabetes Mellitus, CVDs = Cardiovascular Diseases.

Finally, because (visceral) obesity has convincingly been associated with a state of chronic low-grade inflammation, causing a significant strain on healthy immune functioning [19,20], it may, combined with the impact of chronic psychological stress on the immune system, very well close the toxic vicious circle of psychological stress, metabolic and cardiovascular health, and decreased inflammatory resilience [21] (see Figure 1).Glucocorticoids produced by the Hypothalamic—Pituitary—Adrenal (HPA)-axis seem to play a central role in this precarious equilibrium underlying health and well-being [22]. Consequently, effectively investing in cardiovascular fitness might stimulate some highly relevant positive side effects in immune functioning, either directly and/or indirectly via, for example, its effects on mental well-being.

## 2. Exercise Interventions to Relieve the Immune System

Not surprisingly, in case of an inflammatory pandemic such as COVID-19, exercise is considered to be the potentially most cost-efficient and scalable intervention to effectively relieve pressure on the immune system, definitely in anticipation of effective medication or the availability of vaccines, and not least for the most vulnerable individuals [8,23]. This is because on a population scale, relatively small improvements in cardiovascular and respiratory health may have a highly significant effect when it comes to the potential severe complications following (SARS-CoV-2) infection—complications that constitute a significant, global and undeniable strain on healthcare systems. Informing individuals about, and supporting them in, physical activity may therefore be a relevant and useful policy to mitigate at least some of these effects. However, such ambitions are hindered by ambiguity over dose–response relationships. It is yet unclear what type of exercise protocol(s) are most likely to effectively strengthen physical fitness while alleviating the pressure on the immune system, either directly or indirectly (e.g., via its influence on metabolic or psychological processes). That might be confusing and undermines confidence and compliance in the relevant target populations such as the elderly and/or those affected most severely by sedentary behaviors.

Consequently, there seem to be at least 3 fundamental challenges for successful implementation: 1. to be useful, the exercise protocol should be effective within a reasonable and manageable period of time; 2. to stimulate trust and to facilitate the required discipline and motivation for continuation, beneficial effects should manifest themselves overtly and in a timely manner; 3. the protocol should be executable for all ages and social–economic backgrounds [24] by individuals with specific physical states or conditions (i.e., high-risk populations associated with, for example, obesity or cardiovascular syndromes) and under restrained circumstances (e.g., associated with lockdown policies, or in the context of, for example, employability).

### Methods: An Investigative Review

**Figure 2:**
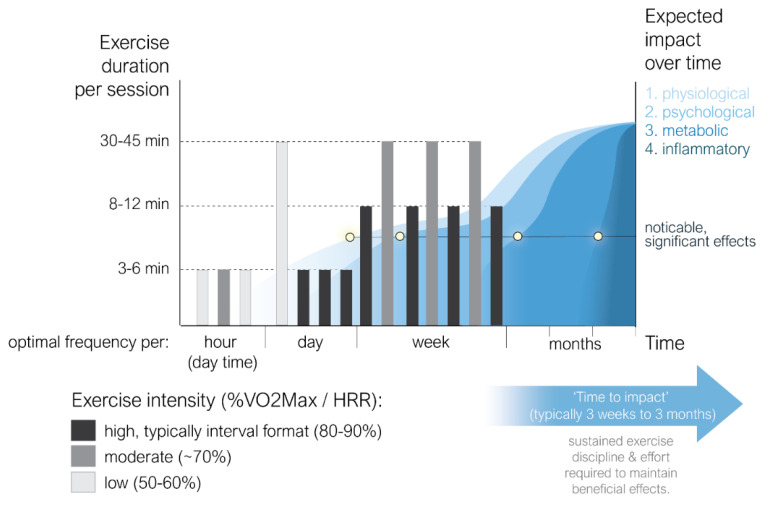
The Summary of the studies covering exercise protocols and their effects on immune functioning. Since metabolic and psychological stress may have a substantial influence on immune processes as well, the reported positive effects of exercise on immune functioning may indirectly be strengthened by its effects via these domains. Measurable effects are reported to typically appear within days (physiological) to months (immune functioning). Note: % VO_2_max = percentage of maximal oxygen uptake, HRR = Heart Rate Reserve.

For these reasons we and others [1,9,21] therefore argue that an active lifestyle is very likely to significantly reduce the risk for complications, potentially already in consecutive waves of SARS-CoV-2 infections, though definitely anticipating the next epidemics and/or pandemics. The present brief investigative review focuses specifically on active lifestyle interventions that could or should work for all ages, keeping a keen eye on the exercise and activity capabilities of mid- to old-age populations. In this way, we aim to address the toxic interactions of sedentary behavior (e.g., prolonged sitting), psychological distress, and immune functioning. Firstly, we present data from recent reviews and meta-analyses, followed by data from individual studies, to provide more detailed information on the actually advised exercise interventions that can be applied in daily life (‘best practices’). Finally, these findings are presented and summarized in one comprehensive exercise protocol that should provide useful guidance for a broad spectrum of target populations (Table 1 and Figure 2).

## 3. Results

### 3.1. Interrupting Prolonged Sitting

A recent meta-analysis reports that regular, relatively frequent light-to-moderate physical activity reduces the postprandial increase in insulin and glucoses, irrespective of the intensity (light or moderate) of the active breaks [25]. Consequently, it may be concluded that the frequency of interruptions may be more relevant than the actual intensity. These findings were confirmed by a second meta-analysis that reports, over 37 included studies (age range 22.1–70.5 years; over one-thousand subjects), a ‘moderately attenuating’ effect of physical activity on glucose metabolism. An effect that seemed to be even more significant with higher BMI and appeared to be stronger following regular ‘breaks’, as compared to a single, extended, light-to-moderate exercise session [26]. Interestingly, the primary findings of these meta-analyses directly address recently identified relevant biomarkers of deteriorating health in the elderly [23]. And in line with these findings, interrupting prolonged sitting is currently generally advised as a preferred strategy in increasing cardio-respiratory fitness in high-risk populations [9], for example in the prevention and treatment of T2D [27].

#### Examples of Best Practises to Interrupt Prolonged Sitting

Multiple studies have empirically investigated the most effective way to induce a measurable effect of relatively short, but frequent, periods of (light-to-moderate) exercise on cardiovascular and/or metabolic health. A useful and effective format may be presented by Wheeler et al. (2019): after 1 h of sitting, they asked their participants to walk for 30 min. Following, after each 30 min of sitting, the participants exercised mildly intensely for 3 min. This program was continued during the rest of the day. At the end of the day, the blood pressure of the participants was positively influenced [28]. Other studies confirm such findings, for example, with a significant positive effect on both insulin metabolism and decreased blood pressure with 5 min of walking after every 30 min of sitting over a working day [29].

A typical example of a high intensity exercise protocol (High Intensity Training: HIT) might be found in the study of Sperlich et al. (2018), who executed a 6-min exercise protocol after 1 h of sitting: 1 min warming-up, followed by 4 min of jumping in 10–20 s bursts at a maximum intensity, and finally 1 min cooling down. Subsequently, the participants sat down for two hours. After this 3-h program, the circulatory (e.g., heart rate) and metabolic processes (e.g., energy expenditure) had still measurably increased [30]. On the other end, there are convincing arguments that after 1 h of sitting, 10 min of either standing or cycling does not nullify the sitting-induced impairment in vasodilation [31]. Consequently, simply standing up (and remain upright) seems not to be sufficient to induce a favorable effect.

### 3.2. Strengthening the Immune System by Exercising

Several reviews indicate that repeated moderately intense exercise (<70% of individual VO2max or personal Heart Rate Recovery (HRR) index, see Figure 2) may measurably strengthen the immune system, for example by enhancing the antibody responses to a vaccine [32]. A positive effect of moderately intense exercise may also be reflected in the mobilization and activation of Natural Killer (NK: parameter of the innate immune system) and/or T cells (associated with the adaptive immune system) [8]. A relevant finding as within the present scope is that there are strong indications that NK cell functioning might be impaired in obese diabetic patients [33], while according to this study, physical activation may actually reactivate the NK cells, a finding that is suggested to be associated with sympathetic nervous system stimulation [34].

#### Examples of Best Practises to Effectively Address the Immune System by Exercise

After one session of 30 min walking, a mild, but measurable, increase in the number of NK cells was reported [35]. This is supported by the finding that 2.5 km of moderately intense walking seems to cause an increase in both the activity and number of NK cells [36].

Additionally, a distinctive increase in the activity of both T and NK cells was reported after a training program of 25–30 min of moderate exercise 3 times every week for 10 months [37]. Other empirical evidence shows that a moderately intense exercise program continued for 3 years, with two 1 h sessions every week, may result in a significant increase in neutrophil activity [38]. Neutrophils are active in both the innate and adaptive immune system and belong to the most prevalent type of white blood cells in mammals.

More recently, a rejuvenation of neutrophil functioning in older pre-diabetics was found following a 10-week supervised low-volume High Intensity Interval exercise Training program (HIIT) of three 30-min sessions every week [39]. In another study, a total of 9 High Intensity Training (HIT) sessions were conducted in 3 contiguous weeks (3 sessions every week) by older pre-diabetic subjects: in every session they walked up- and downhill, at a significant speed, covering 510 altimeters and a total distance of 5 km. Following these training sessions, a significant increase in the number of CD4+ and naïve CD8+ T cells was reported [40]. Finally, moderately intense exercising during 12 weeks, 3 sessions per week, 30 min per session, was reported to enhance the concentration of immunoglobulin A [41], which interacts between the innate and adaptive immune system [42].

However, as addressed by King and colleagues, high-intensity (interval) training (HI(I)T) programs may, at least initially, be too much for individuals suffering from pre-existing cardio-respiratory conditions [9]. Consequently, a phased strategy starting with interrupting prolonged sitting and curving the sedentary lifestyle trend, followed by steadily increasing exercise intensity and duration, might be the most effective and sustainable intervention policy.

### 3.3. Evidence for the Reduction of (Psychological) Stress by Exercising

Chronic (low grade) inflammation, as observed in, e.g., (visceral) obesity [21], may be part of the pathogenesis of mood disorders such as stress, depression, and anxiety [43]. And from that perspective it may not be surprising that a recent meta-analysis identified psychological stress as contributing significantly to the mortality following viral infections [44]. Mikkelsen and colleagues suggest that the established positive effect of exercise on mood is likely related to changes in cytokine release, reducing visceral fat mass, increased vagal tone, and adaptation of the receptors in the immune system [43]. Moreover, an increase in T cells was reported to coincide with a reduction of self-reported chronic stress following moderate exercise [45]. Summarizing: although recent findings clearly indicate that, following extended periods of prolonged sitting, physical activity does not independently affect feelings of mental energy or fatigue [18], overall a physically active lifestyle (regular moderate-to-vigorous physical activities and/or routinely interrupting prolonged sitting) may nevertheless reduce the risk of infectious-disease mortality, which may to some extent be associated with the positive influence that regular moderate exercise has on the relief of psychological distress (see Figure 1 and Table 1).

#### Examples of Best Practises to Stimulate Mental Well-Being by Physical Activity and Exercise

A general and rather straightforward dose–response relationship has earlier been reported between physical activity and the reduction of psychological strain [46].

- A minimum of 1 to 3 times 20 min of low-to-moderate physical activity every week (e.g., walking, gardening, cycling) seems to suffice to initiate measurable improvements in mood and mental health. Interestingly, more than 3 sessions per week seem not to contribute to a further decline in (self-reported) psychological burden;
- Sports activities (moderate to high intensity) seem to induce positive effects that increase even at a training frequency of 4 or more sessions per week;
- Overall, a daily physical activity of any kind seems to provide the best odds ratios in relation to mental well-being.

## 4. Enhancing Cardiovascular Health to Alleviate the Immune Network

In summary, to increase inflammatory resilience, a substantial number of studies advise interrupting prolonged sitting and starting a moderately intense exercise program. As showed in Table 1, the duration and intensity (typically expressed in % of the maximum O_2_ uptake) may vary per session, but at least 30 min per session and 3 times per week appears to be the minimum to indeed induce measurable (positive) results (see Figure 2). Of note is that, irrespective of duration, exercise seems to have a positive effect on various aspects of the immune system, such as NK cells, T cells, immunoglobulin A, and neutrophils. The latter finding is especially promising as neutrophil functioning declines with age, thus contributing to the pathogenesis of many age-related syndromes, such as T2D, and is explicitly identified as a substantial risk factor for complications after (SARS-CoV-2) infections. This finding may be highly relevant for another reason, as the neutrophil-lymphocyte ratio is established as a marker for subclinical inflammation and is recently acknowledged as a strong risk indicator for complications following SARS-CoV-2 infections, even in the early stages following contamination [47,48].

The outcomes summarized in Figure 2 can be conceptualized as a *Physical Activity to Control the Immune Network (Phaccin) Protocol*: highly frequent (hourly) low-to -moderate, 3- to 6-min periods of exercise (‘interrupt prolonged sitting’), alternating with 30 to 45 min of exercise activities once every day, or approximately once every other day when more vigorous, higher intensity (‘sports’) activities are preferred. From literature, it may be concluded that this exercise protocol should provide the optimal combination in relation to physical fitness, metabolic health, psychological well-being, and eventually inflammatory resilience. Noticeable and/or measurable positive effects are estimated to become overt after a few days (physiological) to 3 months (inflammatory) (see Figure 2).

**Table 1:** Overview of the included formats that aim to either revert sedentary behaviors or promote moderate, vigorous or even intensive physical activity.

Exercise Type	Intensity	Frequency	Duration	Practical Example(s)	Reported Effects	Associated Biomarker(s)	Reported Effect Sizes	Representative Reference(s)
Interrupt prolonged sitting	Light to moderate	Once/twice every hour	3 to 6 min	Strolling, knee bends, stretching, other	Hours/days	Blood pressure, Insulin	Positive quadratic trend (*p* < 0.01)	Wheeler et al. 2019 [28]
	High Intensity Interval	Once every 3 h	6 min	Jogging in place, squats, other	Hours/days	Heart rate, energy expenditure	Cohen’s d ≤ 9.35	Sperlich et al. 2018 [30]
Moderate to vigorous physical activity	Moderate (<70% VO_2_max)	Daily	~30 min	Walking, cycling, gardening *	Weeks	Natural Killer (NK) cells, neurophil activity	~30% increase 1 h post exercise	Nieman et al., 2005 [35]
							~50% increase post exercise	Li et al., 2007 [36]
	Vigorous	Three times a week	~30 min	(Brisk) walking, jogging, cycling, swimming, rowing (machine), other	Three months	Immunoglobulin A	~64% increase after 12 weeks	Klentrou et al., 2002 [41]
		Five times a week	~45 min		6 weeks	NK cell activity	56.9% (±10.5%)	Nieman et al., 1990 [49]
		Three times a week	30–45 min		10 months	T and NK cells	A wide range has been reported over multiple studies	Alack et al., 2019 [37]
		Twice a week	~60 min		Three years	Neutrophil activity	Increasing with age	Yan et al., 2001 [38]
High Intensity **	Interval (HIIT)	Three times a week	~30 min	Sprints, jumping, knee bends, other *	10 weeks ***	Neutrophil functioning	Reactive increase 26%, Cohen’s d = 1.12	Bartlett et al., 2020 [39]
	Extensive (HIT)	Three times a week	45–60 min	(Paced) walking up/downhill, running, cycling, rowing	Three weeks ***	CD4+ and naïve CD8+ T cells	All eta-squared ≥ 0.3	Philippe et al., 2019 [40]

Note: * Positive effects have been reported on psychological well-being and associated endocrine functions; ** High intensity (interval) training (HI(I)T) programs may (initially) be too much for individuals suffering from pre-existing cardio-respiratory conditions; *** Reported in pre-diabetic elderly.

We suggest that with increased fitness and confidence, the protocol can be extended with periodic high-intensity, 8- to 12-min training sessions, advisably in an interval format (alternating short periods of 80–90% HRR, with resting periods at <50% VO_2_max). The potential relevance of this add-on seems to be supported by recently published findings indicating that significant exercise up to 15 min ignites a multitude of beneficial immune, metabolic and cardiovascular responses that naturally recover within a reasonable period of time (~one hour) to a healthy baseline [50]. Consequently, we are confident that the phased exercised format thus evolving should provide optimal results within a reasonable period of time and, importantly, be executable for almost everybody.

## 5. Discussion

In applying an exercise program of any kind, one should realize that high intensity, competitive endurance sports, such as extensive and/or vigorous cycling, long-distance swimming or running marathons, may induce an immediate inflammatory response (mirrored, for example, in significant increases in C-reactive protein and interleukin activity, potentially lasting for hours or even days). Importantly, this response is less likely to appear in moderate aerobic activities (such as moderate-pace jogging, dancing or cycling) [51], which can consequently be advised as the optimal choice.

Additionally, although generally speaking the reported effect sizes may be significant but relatively moderate, on average, small improvements in individual fitness may nevertheless have significant effects on a population level with regard to the development of severe complications after infection.

Finally, the present findings are primarily based on reviews and meta-analyses covering singular relations, for example between exercise and immune functioning, the effects of exercise on psychological stress, and sequentially the impact of psychological stress on immune markers. Consequently, longitudinal studies incorporating these factors in one design seem relevant to shed more light on the reciprocal, combined and interactive effects they may induce on the immune system when stimulated by physical exercise.

## 6. Conclusions

The now generally acknowledged high-risk profile for complications, and even mortality, after a SARS-CoV-2 infection highlights in an unprecedented way the horrifying consequences of the already ongoing, though yet globally relatively ignored, sedentary pandemic. And although well meant, governmental ‘lockdown’ policies typically stimulate precisely the contagious combination of sedentary behaviors, psychological strain and (associated) dietary habits that over the past decades contributed so significantly to our vulnerability for complications following opportunistic infections such as variants of SARS-CoV. There is now sufficient evidence, however, that interrupting prolonged sitting and increasing regular physical activity may subtly but measurably enhance immune functioning, especially in the most vulnerable populations. It consequently provides a healthy and economical opportunity to circumvent the impact of the present (COVID-19) pandemic, as well as epidemics and pandemics yet to come. The *Phaccin* protocol introduced here may consequently be considered a useful framework to achieve these beneficial effects by all, within a reasonable period of time and resources.

## Data Availability

All available data has been provided in the present publication: the meta-analysis has been provided in Table 1 and is summarized in Figure 2.
